# Real-Life Clinical Outcomes of Benralizumab Treatment in Patients with Uncontrolled Severe Asthma and Coexisting Chronic Rhinosinusitis with Nasal Polyposis

**DOI:** 10.3390/jcm13144247

**Published:** 2024-07-20

**Authors:** Eusebi Chiner, María Murcia, Ignacio Boira, María Ángeles Bernabeu, Violeta Esteban, Eva Martínez-Moragón

**Affiliations:** 1 Pulmonology Department, University Hospital of Saint John of Alicante, 03550 Alicante, Spain; echinervives@gmail.com (E.C.); maria.murcia11@goumh.umh.es (M.M.); violeta_er@hotmail.com (V.E.); 2 Pharmacy Department, University Hospital of Saint John of Alicante, 03550 Alicante, Spain; bernabeu_marmar@gva.es; 3 Pulmonology Department, Doctor Peset University Hospital, 46017 Valencia, Spain; evamartinezmoragon@gmail.com

**Keywords:** asthma, benralizumab, polyposis, rhinosinusitis

## Abstract

**Background:** The objective of this study was to evaluate, the clinical benefit of benralizumab in patients with uncontrolled severe asthma associated with chronic rhinosinusitis with nasal polyposis (CRSwNP). **Methods:** The study included patients with uncontrolled severe asthma associated with CRSwNP who started therapy with benralizumab. Pulmonary function, eosinophilia, IgE, comorbidity, changes in the Asthma Control Test (ACT), Asthma Control Questionnaire (ACQ), Visual Analogue Scale (VAS), Quality of Life (AQLQ), VAS (obstruction, drip, anosmia, facial pressure), SNOT-22, decrease or withdrawal of steroids and other medication, hospital admissions and emergency visits were analysed. The FEOS scale and EXACTO were employed in the assessment of response. **Results:** We analyzed 58 patients who completed minimal treatment at 12 months. After treatment with benralizumab, exacerbations were reduced by 82% (*p* < 0.001), steroid cycles by 84% (*p* < 0.001), emergencies visit by 83% *p* < 0.001) and admissions by 76% (*p* < 0.001), improving all the scales for asthma control, (*p* < 0.001). In terms of lung function, differences were observed in FVC% (*p* < 0.001), FEV1% (*p* < 0.001), and FEV1/FVC% (69.5 ± 10 vs. 74 ± 10, *p* < 0.001). In relation to CRSwNP, differences were observed in SNOT-22 (54.66 ± 17 vs. 20.24 ± 9, *p* < 0.001), VAS obstruction (7.91 ± 1 vs. 1.36 ± 1, *p* < 0. 001), VAS drip (7.76 ± 1 vs. 1.38 ± 1, *p* < 0.001), VAS anosmia (7.66 ± 1 vs. 1.38 ± 1, *p* < 0.001) and VAS facial pressure (7.91 ± 1 vs. 1.22 ± 1, *p* < 0.001). The mean FEOS score after treatment was 73 ± 14. A complete response/super response was achieved in 33 patients (57%), good response in 16 (28%) and partial response in 9 (15%). **Conclusions:** The administration of benralizumab to patients with uncontrolled severe asthma associated with CRSwNP has been demonstrated to improve nasal symptoms, asthma control and lung function. This resulted in a reduction in the need for oral steroids, maintenance and rescue medication, emergency room visits, and hospital admissions, with 57% of patients achieving the clinical remission criteria.

## 1. Introduction

Despite improvements in the diagnosis and treatment of asthma, approximately 5–10% of patients develop a severe form of the disease, of whom 50% are considered uncontrolled [1,2].

Chronic rhinosinusitis (CRS) affects the nasosinusal mucosa, which may or may not present with nasal polyposis (NP). This is characterised by chronic inflammation with hyperplasia of the sinus mucosa into the nasal cavity. Up to 40% of patients with uncontrolled severe asthma associate chronic rhinosinusitis with nasal polyposis (CRSwNP), with higher severity, more exacerbations and poorer quality of life compared to those without polyposis [3,4].

These two disorders frequently coexist on a common anatomical, immunological, histopathological and pathophysiological basis. Typically, type 2 (T2) inflammation, defined by augmented tissue and peripheral blood eosinophil levels, is the underlying mechanism in asthma with CRSwNP. This process is mediated by T lymphocytes (predominantly T helper type 2), innate lymphoid cells (ILC2) and mast cells, which activate the production of interleukin-5 (IL-5) and the proliferation and differentiation of its receptor (IL-5R). The IL-5R receptor is a heterodimer comprising an IL-5Rα ligand-specific α-subunit and a β-subunit that is common to other cytokine receptors [5].

The differentiation of asthma phenotypes has gained increasing importance due to the progress in individualized treatment approaches and the emergence of biological agents. T2 asthma is triggered by allergens, pollutants, and microorganisms that are captured by dendritic cells leading to the release of interleukin (IL)-25, IL-33, and thymic stromal lymphopoietin (TSLP) from bronchial epithelial cells. These cytokines activate type 2 innate lymphoid cells (ILC2), which play a crucial role in initiating type 2 immune responses. Elevated levels of T helper 2 cell (Th2)-related cytokines such as IL-4, IL-5, IL-13 along with immunoglobulin E (IgE) characterize T2 asthma. Non-T2 asthma lacks clear biomarkers [1].

The discovery of the molecular mechanisms involved in the pathogenesis of asthma has enabled the development of anti-eosinophilic biologic therapies targeting IL-5 (mepolizumab and reslizumab) or its receptor (benralizumab). Dupilumab is a kind of humanized monoclonal antibody that specifically targets the α subunit of the IL-4 and IL-13 receptors, effectively inhibiting the immune effects mediated by IL-4 and IL-13. Tezepelumab’s role in management of type-2 low asthma and potentially non-type 2 asthma is an exciting prospect, however, further studies need to be conducted to explore the mechanisms of these improvements and determine the efficacy of treatment. These therapies have been shown to be efficacy in patients with uncontrolled severe asthma, improving quality of life by reducing exacerbations, improving lung function and reducing systemic corticosteroids (SCS) consumption. Furthermore, they have a good safety profile [6,7].

Benralizumab is a humanised monoclonal antibody that binds to the α-subunit of the IL-5 receptor through its Fab domain, thereby competing against IL-5 binding to its receptor. Furthermore, the afucosylated Fc domain enables binding to the Fc region of the RIIIa receptor on NK cells, macrophages and neutrophils. This results in the activation of cellular cytotoxicity directed at eosinophils, which induces their apoptosis and causes a near-complete depletion [8]. This depleting effect has also been confirmed in induced sputum in a real-life study in which 85% of patients presented a sputum eosinophil count of <3% after 6 months of treatment. [9].

Given that suboptimal control of CRSwNP is associated with reduced asthma control and quality of life, there is a need for the development of new therapeutic approaches. A systematic review evidenced that dupilumab, mepolizumab and benralizumab produced sustained long-term improvement in smell in patients with CRSwNP [10].

Benralizumab has been demonstrated to be effective in the control of uncontrolled severe asthma associated with CRSwNP. A randomised, double-blind, placebo-controlled trial, despite a small sample size, suggested a positive effect in the treatment of CRSwNP with benralizumab. This was evidenced by a statistically significant reduction in nasal polyp size, sinus occupancy, symptoms and improved sense of smell in the majority of patients (83%) [11]. In another study, quality of life and lung function were significantly improved in asthmatic patients with CRSwNP compared to patients with asthma without polyposis [12]. Furthermore, in the CALIMA study, it was concluded that the association of T2 asthma with NP was a predictor of response to anti-IL-5 antibodies in asthma [13]. The phase III OSTRO evaluated the efficacy of benralizumab in symptomatic CRSwNP despite intranasal corticosteroid therapy and a history of CRS or NP surgery evidenced a reduction in nasal obstruction and improvement in olfaction compared to placebo in patients with CRSwNP [14]. Similarly, the subgroup of patients in the ANDHI study with nasal polyps and asthma treated with benralizumab developed an improvement in both asthma and 22-item Sino-Nasal Outcome Test (SNOT-22), with greater improvements observed in patients with higher scores (greater NP involvement) [15].

The objective of this study was to evaluate, in real life, the clinical benefit of benralizumab in patients with uncontrolled severe asthma associated with CRSwNP. Specifically, we assessed the impact of benralizumab on nasal obstruction parameters, as well as on the reduction of hospitalisations, emergency room visits, asthma control and lung function. In addition, we aimed to estimate the clinical remission of severe asthma in patients with CRSwNP-associated asthma after treatment.

## 2. Materials and Methods

### 2.1. Type of Study

A single-centre, observational, prospective and real-life study was conducted on patients with uncontrolled severe asthma who initiated treatment with benralizumab through a joint dispensing protocol with a hospital pharmacy service.

### 2.2. Study Period

January 2019 to September 2023.

### 2.3. Population

The study included all patients ≥ 18 years old with eosinophilic uncontrolled severe asthma and CRSwNP. who were prescribed benralizumab. Uncontrolled severe asthma was defined as a condition that necessitates high-dose inhaled corticosteroids (ICS)/long-acting ß2-adrenergic agonists (LABA) or adjunctive medications such as leukotriene modulators and oral corticosteroids (OCS) for adequate control [1,3].

The following sociodemographic characteristics were assessed: age, sex, body mass index (BMI), smoking status, age of asthma onset, and atopy. Additionally, the levels of immunoglobulin E (IgE) and other biologics previously administered for un-controlled severe asthma were evaluated.

### 2.4. Inclusion Criteria

All patients met the criteria for uncontrolled severe asthma with CRSwNP and received 30 mg benralizumab subcutaneously every 4 weeks for the first 3 doses and then every 8 weeks, starting at T0, for at least 12 months (T12). All patients were clinically assessed in their usual visit regimen at periods between 3 and 6 months between the baseline visit (T0) and the final evaluation visit (T12). All patients had eosinophil counts of ≥150 cells per microlitre (μL) with maintenance treatment with oral glucocorticoids or a history of at least 300 eosinophils/μL in the previous 12 months. Benralizumab was administered when patients had at least two exacerbations in the year prior to onset and uncontrolled severe asthma symptoms.

Exacerbations were defined as a loss of control requiring the administration of oral glucocorticoids for at least three days and/or emergency department visits and/or hospitalisations and/or primary care consultations due to respiratory symptoms. In patients on maintenance oral glucocorticoids, exacerbation was defined as a doubling of the maintenance steroid dose for three days. All patients received a combination of high-dose inhaled corticosteroids (IGCs) and a long-acting beta agonist (LABAs) or a triple regimen of IGCs, LABAs and long-acting antimuscarinics (LAMAs), which could include a leukotriene inhibitor. All patients used an additional controller (short-acting β2-agonists [SABA]) on demand.

CRSwNP was diagnosed by an ear, nose and throat (ENT) specialist in accordance with the Spanish Consensus on the Management of Chronic Rhinosinusitis with Nasal Polyps (POLINA Guide 2023) as the presence of two or more symptoms, one of which must be nasal congestion and/or anterior/posterior rhinorrhoea, plus facial pain/pressure or reduced/loss of smell, for more than 12 weeks [16]. Furthermore, endoscopic signs of nasal polyps and/or mucopurulent discharge and/or oedema in the middle meatus and/or computed tomography (CT) changes of the middle meatus or sinuses were observed.

All patients underwent nasal examination, endoscopy and sinus computed tomography (CT) when appropriate and had a history of sinus surgery. All patients were using IGC and nasal antihistamines.

### 2.5. Efficacy Monitoring

The assessment of asthma symptom control, oral glucocorticoid dose, exacerbations and forced expiratory volume in 1 s (FEV1) was conducted at baseline and at scheduled visits at 6 and 12 months after baseline. Spirometry was performed in accordance with the Spanish Society of Pulmonology and Thoracic Surgery (SEPAR) criteria [17], with reversibility testing conducted in all patients prior to benralizumab initiation.

The self-administered Asthma Control Test (ACT) [18] and Asthma Control Questionnaire (ACQ) [18] were employed to monitor symptoms at baseline and throughout the course of treatment, while the mini–Asthma Quality of Life Questionnaire (miniAQLQ) was utilized to assess quality of life.

The SNOT-22 questionnaire [19] was used to assess the response in CRSwNP. SNOT-22 was developed for use in chronic rhinosinusitis and assesses the symptoms and functional and emotional consequences of chronic rhinosinusitis through responses to 22 items using a 6-category scale, from 0 (no problem) to 5 (problem as bad as it can be). Greater scores indicate a poorer outcome (range 0–110).

A visual analogue scale (VAS) was also employed to assess nasal obstruction, rhinorrhea, anosmia, and facial pressure. The scale ranged from 0 to 10, with 0–3 being classified as mild, >3–7 moderate, and >7–10 severe. The higher the score, the greater the subjective burden of CRSwNP symptoms, and the lower the score, the lesser the burden [16].

The response to biological treatment between T0 and T12 was evaluated using the FEOS scale [20] and the degree of asthma control using the multidimensional EXACTO scale, which classifies patients into four categories: non-response, partial response, good response, and complete response/super-responder, based on exacerbations, ACT, oral glucocorticoids, and FEV1 according to changes from T0 [21].

### 2.6. Statistical Study

A statistical study was conducted to analyse the data. The baseline or outcome numerical variables were presented as mean and standard deviation or as median and interquartile range. All differences were assessed by comparing values at T0 with T12 using either the Student’s *t*-test (for paired data) or the Wilcoxon test, depending on the normality of the data. A *p*-value of less than 0.05 was considered statistically significant for all parameters recorded. All statistical analyses were conducted using SPSS version 18.

### 2.7. Ethical Considerations

The study was conducted in accordance with the ethical principles. Written informed consent was obtained from all patients included in the study. The study was approved by the ethics committee.

## 3. Results

Fifty-eight patients were analysed in the study, all of whom completed treatment with benralizumab at 12 months. Of the patients studied, 6 (10%) had previously been treated with omalizumab. The baseline characteristics of the patients are shown in Table 1.

**Figure 1:**
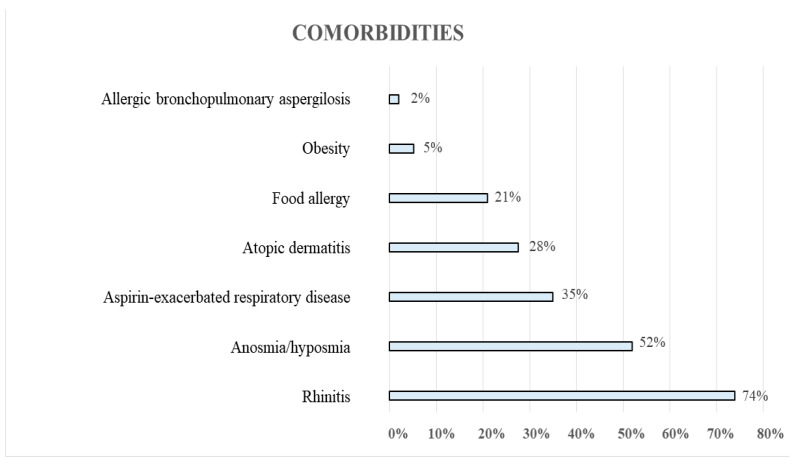
Comorbidities of the patients under study.

An overall 93% of patients had at least one comorbidity, with rhinitis being the most frequent (Figure 1).

**Figure 2:**
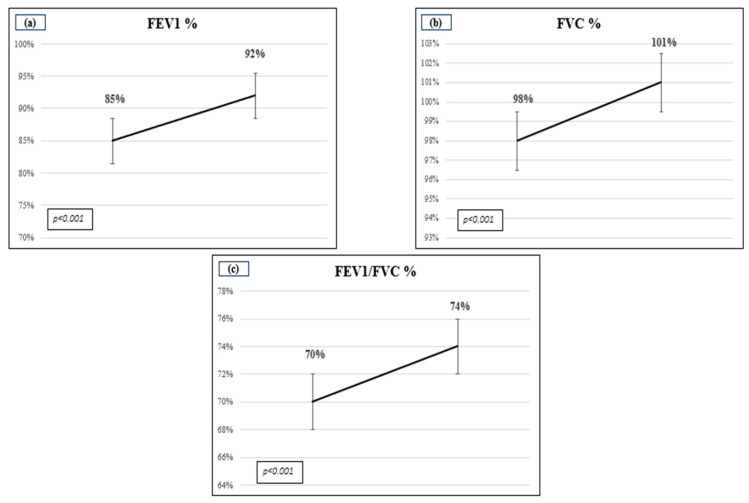
A comparison of respiratory function before and after treatment. (**a**) FVC% (Forced Vital Capacity expressed in %); (**b**) FEV1% (Forced Expiratory Volume in the 1st second expressed in %) and (**c**) FEV1/FVC ratio in %.

Twenty-two patients (37.9%) were identified as current smokers. The treatment administered to patients consisted of one or more drugs. All patients were prescribed high-dose IGCs, 98% LABAs, 88% SABAs, 45% montelukast, 45% LAMAs, 21% antihistamines, 57% anti-leukotrienes, 1.7% xanthines and 5% maintenance oral glucocorticoids. In total, 76% of patients received nasal corticosteroids in combination with local and/or systemic antihistamines. In terms of lung function, a comparison of baseline and after treatment values revealed statistically significant improvements in FVC%, FEV1% and FEV1/FVC% (Figure 2).

**Figure 3:**
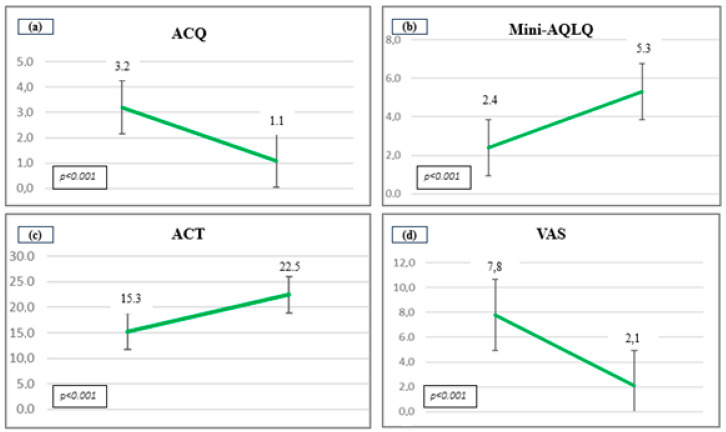
Changes in punctuation of the scales after the treatment. (**a**) ACQ (Asthma Control Questionnaire); (**b**) Mini-AQLQ (Asthma Quality of Life Questionnaire); (**c**) ACT (Asthma Control Test) and (**d**) VAS (Visual Analogue Scale).

Moreover, significant changes were also observed in punctuation of ACQ (3.19 ± 1 vs. 1.1 ± 0.81, *p* < 0.001), ACT (15.33 ± 1.59 vs. 22.48 ± 2, *p* < 0.001), mini-AQLQ (2.4 ± 0.38 vs. 5.3 ± 0.84, *p* < 0.001) and VAS (7.57 ± 1 vs. 2.1 ± 1, *p* < 0.001) and VAS (7.57 ± 1 vs. 2.1 ± 1, *p* < 0.001) (Figure 3).

With regard to CRSwNP, significant differences were observed in the punctuation of SNOT-22 after the treatment (55 ± 17 vs. 20 ± 9, *p* < 0.001). Furthermore, there was a significant improvement in VAS obstruction, VAS drip, VAS anosmia and VAS facial pressure (8 ± 1 vs. 1 ± 1, *p* < 0.001).

We compared post-treatment SNOT-22 response by groups with response < 40 points or a difference of less than 12 points from baseline, according to the presence of atopy or not, and by eosinophil level < 500 cells/μL or greater than 500 cells/μL.

In this regard, 35 patients (60%) had negative and 23 (40%) positive pricks or RAST and 16 patients (29%) had less than 500 eosinophils and 42 (71%) more than 500 eosinophils). When comparing the two groups, 8 patients (15%) had a score over 40 post treatment, of which 7 (30%) were atopic and 1 (3%) non-atopic (chi square 4.705, *p* = 0.032), indicating a better response in non-atopic patients.

Regarding eosinophils, with less than 500 cells/μL, 9 (56%) presented change in SNOT-22 < 12 versus 6 (14%) in the group with more than 500 cells/μL (chi square 4.214, *p* = 0.04), indicating better response with greater eosinophilia.

**Figure 4:**
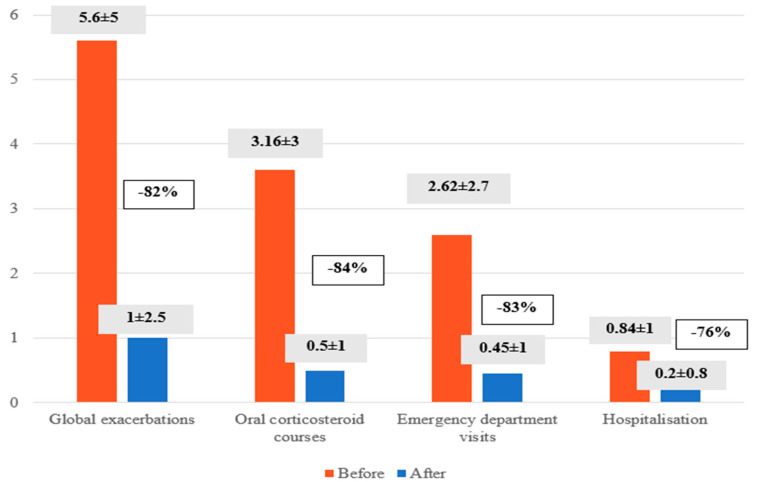
A comparison of the incidence of global exacerbations, the number of oral corticosteroid courses administered, the number of emergency department visits and the number of hospitalisations before and after benralizumab treatment.

In the year prior to the initiation of benralizumab, 33% of patients had been hospitalised, 70% had visited the emergency department, and 72% had received courses of oral glucocorticoids. The mean number of exacerbations per patient per year was 5.6 ± 5. After treatment with benralizumab, exacerbations were reduced by 82% (5.6 ± 5 vs. 1 ± 2.5, *p* < 0.001), the number of steroid cycles by 84% (3, 16 ± 3 versus 0.5 ± 1, *p* < 0.001), emergency department visits by 83% (2.62 ± 2.7 versus 0.45 ± 1, *p* < 0.001) and hospital admissions by 76% (0.84 ± 1 versus 0.2 ± 0.8, *p* < 0.001) (Figure 4).

**Figure 5:**
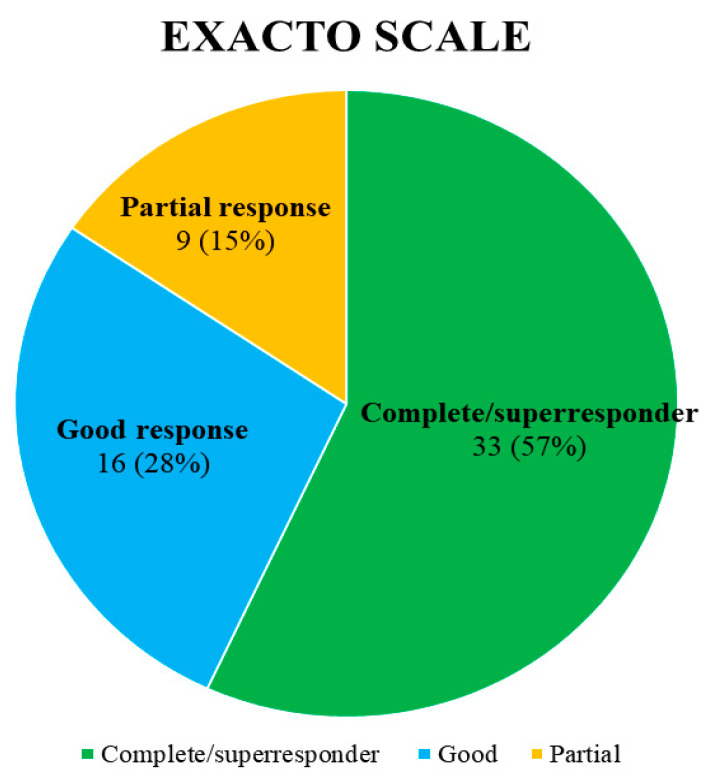
Consensus response EXACTO (Exacerbations, ACT, systemic corticosteroids and FEV1-Obstruction) to benralizumab treatment: partial response, good response or complete/superresponder.

The mean FEOS score after treatment was 73 ± 14. According to the multidimensional EXACTO scale, 33 patients (57%) achieved a complete or super-response, 16 (28%) a good response, and 9 (15%) a partial response (Figure 5).

The adverse effects observed were minor: transient fever in 5 patients, odynophagia in 4, headache in 2, with no major adverse effects requiring discontinuation of treatment.

## 4. Discussion

The coexistence of uncontrolled severe asthma and CRSwNP has been demonstrated to exert a deleterious influence on asthma control and quality of life. This highlights the necessity for the development of novel additional treatment options to provide adequate control of uncontrolled severe asthma associated with CRSwNP [1].

In the case of CRSwNP, the POLINA guidelines recommend the use of individual or global assessment scores of nasal symptoms to measure the response to treatment. These may be measured using a VAS or a semi-qualitative score, as well as a register of exacerbations and measurement of SNOT-22. In contrast, with regard to uncontrolled severe asthma, the Spanish Guideline on the Management *of* Asthma (GEMA) indicate that the response to treatment should be evaluated based on the number of exacerbations, the reduction or withdrawal of pharmacological treatment necessary for control (especially oral glucocorticoids), subjective asthma control rating scales (ACT, ACQ, VAS and AQLQ), and the impact on lung function as measured by FEV1 [1]. These criteria have been evaluated in this study and found to be comparable to those of published trials such as CANONICA [2] and OSTRO [14], as well as other real-life studies [22,23].

The characteristics of the patients in our study were similar to those of recently published studies. Specifically, our population had a mean age of 56 years and a higher percentage of women, as in OSTRO [14] and CANONICA [2], as well as elevated levels of eosinophils and IgE. This is consistent with the findings of the aforementioned studies, which suggest that eosinophils may play an important role in the pathogenesis of uncontrolled severe asthma with CRSwNP, representing a potential therapeutic target. With regard to lung function, the mean FEV1 was found to be compatible with obstructive pathology, and ACQ was elevated, indicating poor asthma control. It was noteworthy that a high proportion of patients were receiving oral corticosteroids and that there was a significantly increased annual exacerbation rate and SNOT-22.

In relation to uncontrolled severe asthma, our study demonstrated a statistically significant improvement in lung function, subjective asthma control and quality of life, as well as a reduction in the annual global exacerbation rate of 82%. These findings are comparable to those of the CANONICA study, which observed a 0.5 L improvement in FEV1, a reduction in ACQ of 1.69 points, and a significant reduction in annual asthma exacerbations [2]. With regard to the response of asthma to biologic therapy, a notably high score was achieved on the FEOS scale, as well as on the multidimensional EXACTO scale. Furthermore, in terms of clinical remission, 57% of patients exhibited a complete response or super-response. These results are comparable to those obtained in a recently published article on the response and remission of uncontrolled severe asthma with biologic therapy [24]. With respect to CRSwNP, a reduction of 25 points was observed on the SNOT-22 scale, with a mean value of 20 points after treatment. This is higher than that shown in studies such as OSTRO (−6.23 points) [14] and similar to that of CANONICA (−20 points) [2]. Although the POLINA guidelines do not establish a specific SNOT-22 value to be considered a response to treatment with a biologic, most authors use a reduction of 8.9 points with respect to baseline and SNOT-22 below 30 points as an important variation in terms of response to treatment, which is consistent with the findings of our study [2,16,25]. In our study, patients with a higher degree of eosinophilia and a lower component of atopy presented better nasal response, although without differences in asthma control.

Patients with uncontrolled severe asthma typically require treatment with oral glucocorticoids, which, in the case of association with CRSwNP, may be increased and persistent. Given that long-term administration of oral glucocorticoids may lead to adverse effects, it is important to reduce their consumption. Regarding to the impact of benralizumab on oral corticosteroid consumption, the ZONDA trial demonstrated that benralizumab administration resulted in a 75% reduction in oral glucocorticoids use [26]. The results demonstrated a more favourable outcome, with a reduction of 84% in the maintenance oral glucocorticoid use compared to those observed in the ANANKE real-life study, where a 64% reduction in oral glucocorticoid use was observed [22].

An essential aspect of our study is that it is a real-life study, which allows us to complement the information collected in the literature to date. This is because our work includes a heterogeneous group of patients who have been followed and treated in a way that is adjusted to daily clinical practice. In our case, the study included a large number of patients (n = 58) collected in a single centre and with a uniform work system throughout the follow-up of patients during the study, which can be interpreted as a strength.

This can be compared with other multicentre real-life studies. On the one hand, two Italian studies with a small patient sample: one comprising 59 patients with uncontrolled severe asthma of which 34 had CRSwNP [27] and another with 10 patients with uncontrolled severe asthma and CRSwNP [28]. Additionally, there are larger-scale real-life studies with greater patient numbers, such as the ANANKE study with 110 patients [22], Nolasco et al. with 79 patients [29] and Pini et al. with 108 patients [30]. Despite the differences in the samples, all of the studies presented similar results and conclusions regarding the efficacy of benralizumab in patients with uncontrolled severe asthma and CRSwNP.

Given that the results of our study show a very significant efficacy in the control of uncontrolled severe asthma NCSA and associated CRSwNP, other published clinical trials such as SIROCCO [31] and CALIMA [13], as well as real-life studies comparing the efficacy of benralizumab in asthma patients with and also agree that nasal polypo-sis is a consistent predictor of response to benralizumab in terms of asthma control outcomes, with better outcomes in patients with associated CRSwNP. Additionally, benralizumab has shown to be demonstrated more cost-effective (52.21 quali-ty-adjusted life years [QALYs]) than mepolizumab (51.39 QALYs) and dupilumab (51.30 QALYs) [32].

Limitations of our study include the fact that the only information we have on CRSwNP in patients is their diagnosis by an ENT specialist, although we do not have precise information on imaging techniques before and after the study, but the number of previous nasal surgical treatments.

ENT specialist also work in real life and the study was not designed as a prospective study including all objective data such as imaging techniques or smell tests but as a clinical study carried out in routine clinical practice and this should be taken into con-sideration.

In conclusion, benralizumab demonstrated efficacy in improving nasal symptoms as measured by SNOT-22, as well as in reducing obstruction, drip, anosmia and facial pressure parameters. Furthermore, it was associated with a significant reduction in the number of hospital admissions and emergency department visits, as well as a reduction in oral corticosteroid consumption. Additionally, benralizumab was associated with improved lung function in patients with uncontrolled severe asthma with CRSwNP, with two-thirds of patients achieving complete asthma remission.

## Figures and Tables

**Table 1:** General characteristics of the patients included in the study (n = 58). BMI: Body Mass Index. IgE: Inmunoglobulin E. FEV1: forced expiratory volume in 1 s. FVC: Forced Vital Capacity. ACT: Asthma Control Test. ACQ: Asthma Control Questionnaire. VAS: Visual Analogue Scale. SNOT-22: 22-item Sino-Nasal Outcome Test. AQLQ: Asthma Quality of Life Questionnaire. ENT: Ear, Nose and Throat.

Baseline Characteristics	Value/Mean ± Standard Deviation
Mean age (years)	55 ± 14
BMI (Kg/m^2^)	27 ± 8
Sex	64% women, 36% men
Eosinophils, cel/μL	786 ± 504
IgE (UI/mL)	361 ± 807
Atopy/allergy	17 patients
FEV1 (Liters and %)	2.45 ± 0.8 (84 ± 18%)
FEV1/FVC (%)	69.5 ± 10 (%)
FVC (liters and %)	3.47 ± 1.01 (97 ± 16%)
ACT // ACQ// VAS	15 ± 2 // 3 ± 1 // 7.5 ± 1
SNOT-22	55 ± 17
VAS:	
Obstruction	8 ± 1
Runny nose	7.8 ± 1
Anosmia	7.6 ± 1
Facial pressure	7.8 ± 1
Mini-AQLQ	2.4 ± 0.4
Number emergency visits / year	2.62 ± 2.7
Number of hospital admissions/year	0.84 ± 2
Days of hospital admission	3 ± 8
Global exacerbations	5.6 ± 5
Oral glucocorticoid cycles	3.16 ± 3
Time of treatment (months)	14.4 ± 8
Previous ENT surgery (one or more)	Total: 55% (32) One: 47% (15) Two: 31% (10) Three or more: 22% (7)

## Data Availability

Data are contained within the article.
